# Computational Modeling of the Staphylococcal Enterotoxins and Their Interaction with Natural Antitoxin Compounds

**DOI:** 10.3390/ijms19010133

**Published:** 2018-01-03

**Authors:** Mahantesh Kurjogi, Praveen Satapute, Sudisha Jogaiah, Mostafa Abdelrahman, Jayasimha Rayalu Daddam, Venkatesh Ramu, Lam-Son Phan Tran

**Affiliations:** 1 Plant Healthcare and Diagnostic Center, Department of Studies in Biotechnology and Microbiology, Karnatak University, Dharwad 580003, India; mahantesh.kurjogi@gmail.com (M.K); psatapute6@gmail.com (P.S.); 2 Department of Botany, Faculty of Sciences, Aswan University, Aswan 81528, Egypt; meettoo2000@ige.tohoku.ac.jp; 3 Graduate School of Life Sciences, Tohoku University, 2-1-1 Katahira, Aoba-ku, Sendai 980-8577, Japan; 4 Department of Biotechnology, Jawaharlal Nehru Technology University, Anantapur 515002, India; jayasimharayalu@gmail.com; 5 Department of Biochemistry, Indian Institute of Science, Bengaluru 560012, India; venka.biotech@gmail.com; 6 Institute of Research and Development, Duy Tan University, 03 Quang Trung, Da Nang 550000, Vietnam; 7 Signaling Pathway Research Unit, RIKEN Center for Sustainable Resource Science, 1-7-22 Suehiro-cho, Tsurmi-ku, Yokohama 230-0045, Japan

**Keywords:** *Staphylococcus aureus*, enterotoxin, food poisoning, in silico, Betulin, 28-Norolean-12-en-3-one, 3-D structure, amino-acid residues, docking

## Abstract

*Staphylococcus aureus* is an opportunistic bacterium that produces various types of toxins, resulting in serious food poisoning. Staphylococcal enterotoxins (SEs) are heat-stable and resistant to hydrolysis by digestive enzymes, representing a potential hazard for consumers worldwide. In the present study, we used amino-acid sequences encoding SEA and SEB-like to identify their respective template structure and build the three-dimensional (3-D) models using homology modeling method. Two natural compounds, Betulin and 28-Norolean-12-en-3-one, were selected for docking study on the basis of the criteria that they satisfied the Lipinski’s Rule-of-Five. A total of 14 and 13 amino-acid residues were present in the best binding site predicted in the SEA and SEB-like, respectively, using the Computer Atlas of Surface Topology of Proteins (CASTp). Among these residues, the docking study with natural compounds Betulin and 28-Norolean-12-en-3-one revealed that GLN43 and GLY227 in the binding site of the SEA, each formed a hydrogen-bond interaction with 28-Norolean-12-en-3-one; while GLY227 residue established a hydrogen bond with Betulin. In the case of SEB-like, the docking study demonstrated that ASN87 and TYR88 residues in its binding site formed hydrogen bonds with Betulin; whereas HIS59 in the binding site formed a hydrogen-bond interaction with 28-Norolean-12-en-3-one. Our results demonstrate that the toxic effects of these two SEs can be effectively treated with antitoxins like Betulin and 28-Norolean-12-en-3-one, which could provide an effective drug therapy for this pathogen.

## 1. Introduction

*Staphylococcus aureus* is among the most common microflora present on the skin and mucous membrane of humans and cattle [1]. Despite its generally benign nature, *S. aureus* can turn opportunistic and become pathogenic. The pathogenic nature of *S. aureus* has been widely reported in cattle, which was found to be the predominant cause of bovine mastitis [2]. The induction of intramammary infection by *S. aureus* requires the production of a variety of virulence factors secreted by the pathogen needed for adherence, colonization and invasion of epithelial cells of the mammary glands of the cows [3]. In addition to its opportunistic pathogenicity, *S. aureus* is the most prevalent enterotoxin producing microbe that causes food-borne diseases among *Staphylococcus* species worldwide [4]. The possession of virulence factors and survival of *S. aureus* in the host environment are attributed to the self-defense mechanism expressed by *S. aureus* that helps in protection against defense molecules produced by the host [5,6].

*Staphylococcus* spp. secret many potential virulence factors, or exotoxins, such as α- and β-toxins, toxic shock syndrome toxin and enterotoxins [7]. Both α- and β-toxins cause lysis of erythrocytes by pore formation or disrupting normal cellular metabolism [8,9]. Staphylococcal enterotoxins (SEs) are responsible for major food poisoning outbreak with symptoms, such as violent vomiting, nausea and abdominal cramping with or without diarrhea [10,11,12,13]. In growth curve, these toxins are synthesized during the transition from logarithmic phase to the stationary phase, highly active even at low concentrations and resistant to heat as well as proteolytic enzymes [14,15]. Therefore, enterotoxins are active even in the presence of host digestive enzymes [16,17,18]. In addition to their gastrointestinal effects, enterotoxins also cause pyrogenic immune suppression and non-specific T-cell proliferation, and are thus aptly referred to as superantigens [19].

Staphylococcal food poisoning is highly under-reported because of misdiagnosis, minor outbreaks, improper sample collections and false positive lab reporting. In addition, the poor personal hygiene is considered to be one of the critical elements, which causes *S. aureus* infection, making disease control process more complicated. Although there are more than 20 distinct SEs, only a few of them have been examined in depth [4,20]. The SEA and SEB are the most common toxins involved in *Staphylococcus*-related food poisoning followed by SED [20]. However, in spite of the available knowledge on the enterotoxins produced by *S. aureus*, an effective control strategy is still lacking. New and cheaper drugs that target the active sites of the toxins should be designed in order to increase the affordability of drugs against the highly evolving *S. aureus*. In our previous study, we have identified a gene encoding SEB-like in *S. aureus* isolated from milk samples [21]. In this context, the focus of the present study was to construct the three-dimensional (3-D) model structures of our SEB-like and the SEA of *S. aureus* Newman isolated previously from human infection [22], which will serve as useful targets for structure-based drug designing. We have also carried out docking studies with the designated binding sites of the examined SEA and SEB-like using potent natural molecules Betulin and 28-Norolean-12-en-3-one.

## 2. Results and Discussion

### 2.1. Homology Modeling of SEA and SEB-Like Proteins

**Figure 1:**
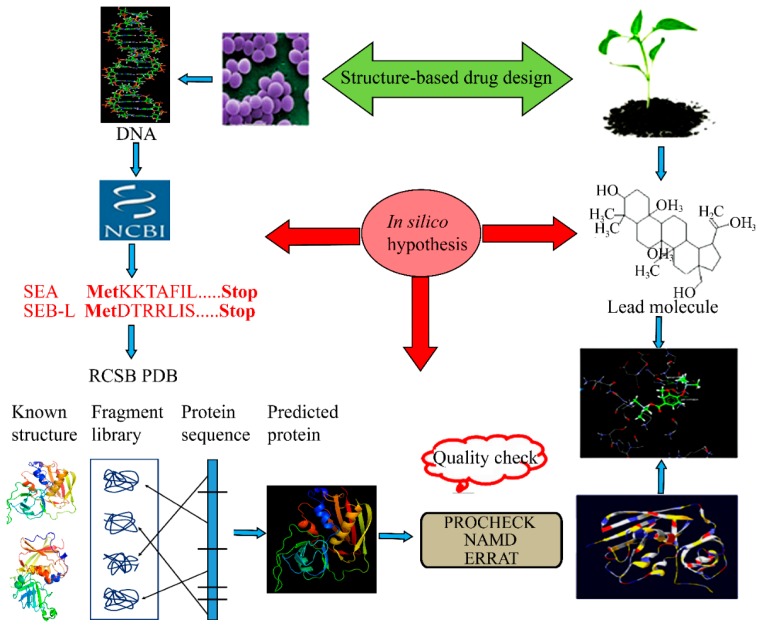
Workflow of structure-based drug design. The amino-acid sequences of staphylococcal enterotoxins A (SEA) and B-like (SEB-L) were obtained from National Center for Biotechnology Information (NCBI). The amino- acid sequences were blast-searched against the Research Collaboratory for Structural Bioinformatics Protein Data Bank (RCSB PDB) to identify the related protein structures that were subsequently used as templates in homology modeling. The quality and reliability of the generated models were assessed by NAMD 2.5 (NAnoscale Molecular Dynamics 2.5), PROCHECK and ERRAT programs.

The present study extends our previous work to develop the 3-D model structure of the SEB-like that we identified from milk samples [21], and that of the SEA previously isolated from *S. aureus* Newman [22]. These 3-D model structures will serve as a useful tool for structure-based drug designing (Figure 1).

**Figure 2:**
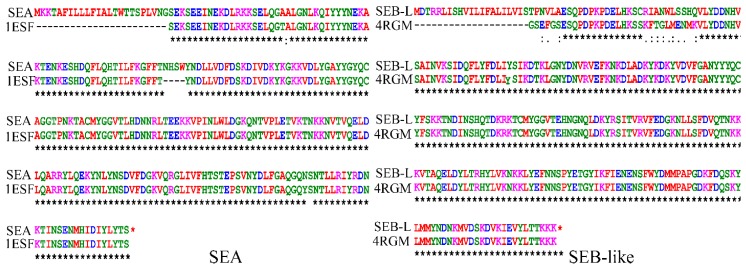
Amino-acid sequence alignments of staphylococcal enterotoxins A (SEA) and B-like (SEB-L) with their template structure sequence. 1ESF is the template structure of SEA, while 4RGM is that of SEB-like.

Homology modeling is a multi-step process consisting of template selection, sequence alignment, alignment correction, model building, optimization and model validation. In the series of homology modeling process, templates were chosen and utilized for further validation studies [23]. Homology modeling cannot be performed automatically as it depends on the percentage of sequence identity of template structure with the query sequence [24]. In the present study, the amino-acid sequences of the SEA and SEB-like were blast-searched against the Research Collaboratory for Structural Bioinformatics Protein Data Bank (RCSB PDB) using default parameters, revealing 1ESF and 4RGM as the template structure for SEA and SEB-like, respectively, based on their maximum identity and minimum *E*-value (Figure 2). The identity and *E*-value between the 1ESF and the SEA were 94% and 3.03 × 10^−124^, respectively, whereas these values were 95% and 1.69 × 10^−132^ between the 4RGM and the SEB-like.

### 2.2. Homology Modeling of the 3-D Structures

**Figure 3:**
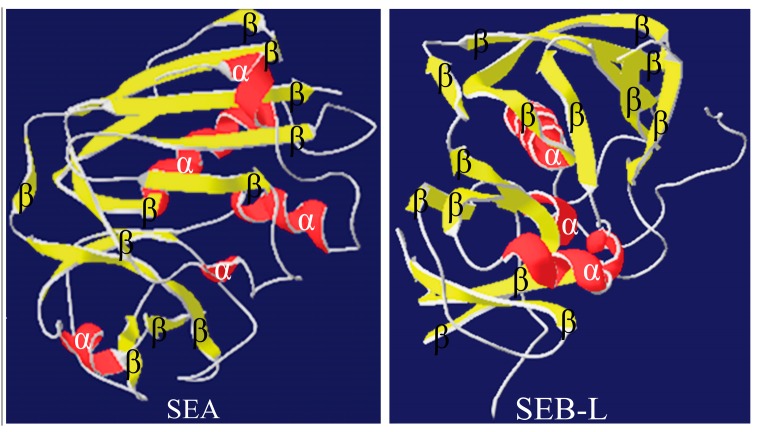
Three-dimensional (3-D) structures of the enterotoxins. The 3-D structure of staphylococcal enterotoxin A (SEA) with five α-helices and 11 β-sheets, and that of staphylococcal enterotoxin B-like (SEB-L) with three α-helices and 14 β-sheets.

The 3-D models of SEA and SEB-like were generated by the MODELLER 9.17 software using the template structures 1ESF and 4RGM (Figure 3). Coordinates of structurally conserved regions C-termini and N-termini from the 1ESF and 4RGM templates, and structurally variable regions were designated to target sequences according to the satisfaction of the spatial restraints. All side chains of the model proteins were set by rotamers. On the basis of the 3-D structures, the valid models of the enterotoxins SEA and SEB-like were selected.

### 2.3. Validation of the 3-D Structures

#### 2.3.1. Molecular Dynamics Studies

**Figure 4:**
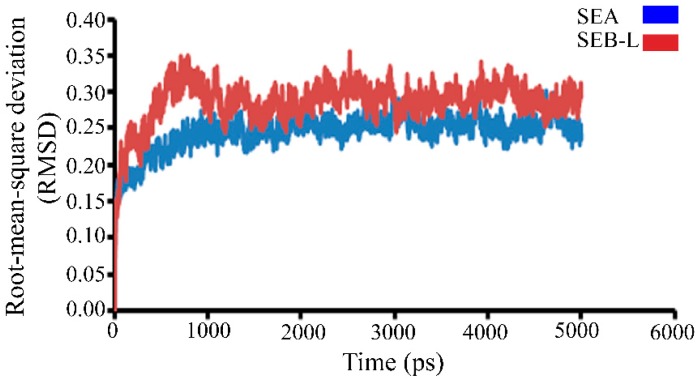
Trajectories of the root-mean-square deviation (RMSD) of carbon backbone trace of staphylococcal enterotoxins SEA (blue) and SEB-like (SEB-L, red) with respect to simulation time in picoseconds (ps).

The stability of the 3-D SEA and SEB-like structures was examined by the variation in total energy versus time. Furthermore, the trajectory graph was also plotted between the root-mean-square deviation (RMSD) of carbon backbone trace (C trace) and time in picoseconds (ps) (Figure 4). The steady value stretches out between 0.1 and 0.35 Å, and RMSD value increased initially and stabilized at 1000 up to 5000 ps, due to lower fluctuation of RMSD values, and can be considered as a measure of the quality and stability of the protein models.

#### 2.3.2. Ramachandran Plot Analysis

**Figure 5:**
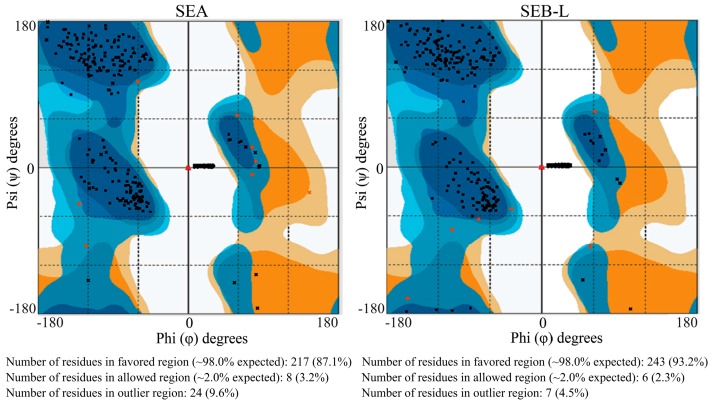
Ramachandran plot for the staphylococcal enterotoxins A (SEA) and B-like (SEB-L). Different colors indicate favored (dark blue and dark brown for non-glycine and glycine residues, respectively) and allowed (light blue and light brown for non-glycine and glycine residues, respectively) regions. Black and red squares indicate general residues including pre-prolines (residues immediately preceding a proline in sequence, except glycine and proline) in favored and allowed region, respectively. Black and red triangles indicate proline residues in favored and allowed region, respectively. Black and red crosses indicate glycine residues in favored and allowed region, respectively.

The energetically allowed and favored regions for SEA and SEB-like backbone dihedral angles Psi (ψ) on the *y*-axis against Phi (ϕ) on the *x*-axis of amino-acid residues were visualized using Ramachandran plot (Figure 5). Each dot indicates the angle for an amino-acid, and the areas on the plot with the highest density of dots are called ‘favored regions’ (Figure 5).

Introduction of a few errors in the homology modeling is a common phenomenon [25], and more attention is needed toward refinement and validation of the structure obtained. All the errors that occurred in homology modeling process were assessed and validated after the refinement of protein structure [25]. The validation of the SEA and SEB-like 3-D models obtained in this study was also carried out using Ramachandran plot, and the corresponding calculations were computed with the RAMPAGE program. The Ramachandran plot results together indicated that 87.1% and 93.2% of the residues of the SEA and SEB-like models, respectively, were in their favored region (Figure 5). The validation results using the Ramachandran plot revealed the stable conformation of constructed models with appropriate stereochemistry, indicating that these models can be used for further analyses. The Ramachandran plot parameters in RAMPAGE program used in our study are probably the most potent determinants for the protein structural evaluation and validation [26,27].

#### 2.3.3. Analysis of the SEA and SEB-Like 3-D Structures

The validation of SEA and SEB-like 3-D structures was further performed using the Structure Analysis and Verification Server. The RMSD values obtained from VERIFY_3D for covalent bonds and covalent angels relative to the standard dictionary value of the SEA model were −0.58 and −2.68 Å, and SEB-like model were −1.78 and −0.39 Å, respectively. The overall PROCHECK G-factor of the SEA and SEB-like models was −3.47 and −1.26 Å, respectively, and the verified 3-D environmental profile was found to be suitable for both models.

Several methods are available for comprehensive evaluation of structural alignment in a model, which include superposition of model into the native structure with the structure alignment program, measurement of the average distance between the backbone atoms of superimposed proteins by the RMSD and generation of the Z-score [28,29]. The evaluation of the model also involves the development of a scoring function that is able to distinguish bad and good models. A variety of statistical criteria have to be derived from various properties, such as distributions of polar residues outside or inside the protein [30]. For error corrections, solvation potential that can detect local errors and complete misfolding in protein structures has to be used [31], and packing rules have to be implemented for structure evaluation [32]. A model is considered valid when only a few distortions are present in atomic contacts.

**Figure 6:**
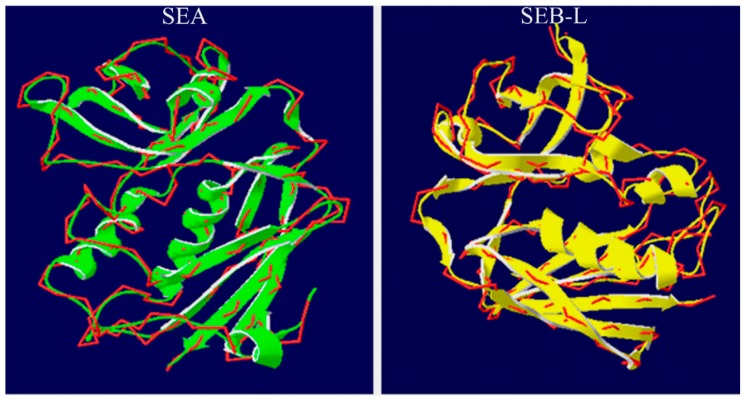
Superimposition of C-α chain (green) of staphylococcal enterotoxin A (SEA) and template 1ESF (red), and of C-α chain (yellow) of staphylococcal enterotoxin B-like (SEB-L) and template 4RGM (red).

After the final SEA and SEB-like 3-D models were built, their possible active pockets were determined using the Computer Atlas of Surface Topology of Proteins (CASTp) [33,34]. CASTp can be used to analyze various conserved regions of proteins, and it provides accessibility to the binding sites on a protein that are associated with the effectiveness of docking [34]. For instance, the CASTp has been used to determine the active pocket in the 3-D structure of the Anthrax Toxin Receptor 1 [24]. We conducted a CASTp analysis based on the volume of pockets, the atoms lining the pockets and the area circumference of mouth openings. A total of 41 and 37 binding sites were identified in the 3-D structure of SEA and SEB-like, respectively. Among these, the best predicted binding site was selected for further docking analysis based on the area and volume of the binding site, which can help in binding the ligand molecule in all directions. The binding site of SEA with an area of 215.2 Å^2^ and a volume of 215.1 Å^3^ had 14 amino-acid residues, including ARG37, LYS38, GLU41, LEU42, GLN43, ALA46, LEU50, PHE223, GLY224, ALA225, GLN226, GLY227, GLN228 and LEU233, whereas that of SEB-like with an area of 240.6 Å^2^ and a volume of 277Å^3^ contained 13 residues, namely VAL53, LEU54, ASN58, HIS59, LEU85, ASN87, TYR88, ALA114, ASN115, TYR116, TYR117, TYR189 and GLN237 (Figure S1). Additionally, we used Swiss-PDB Viewer to scrutinize the structural comparison by superimposition of the template and model, which further strengthens our validation (Figure 6).

### 2.4. Docking Study

**Figure 7:**
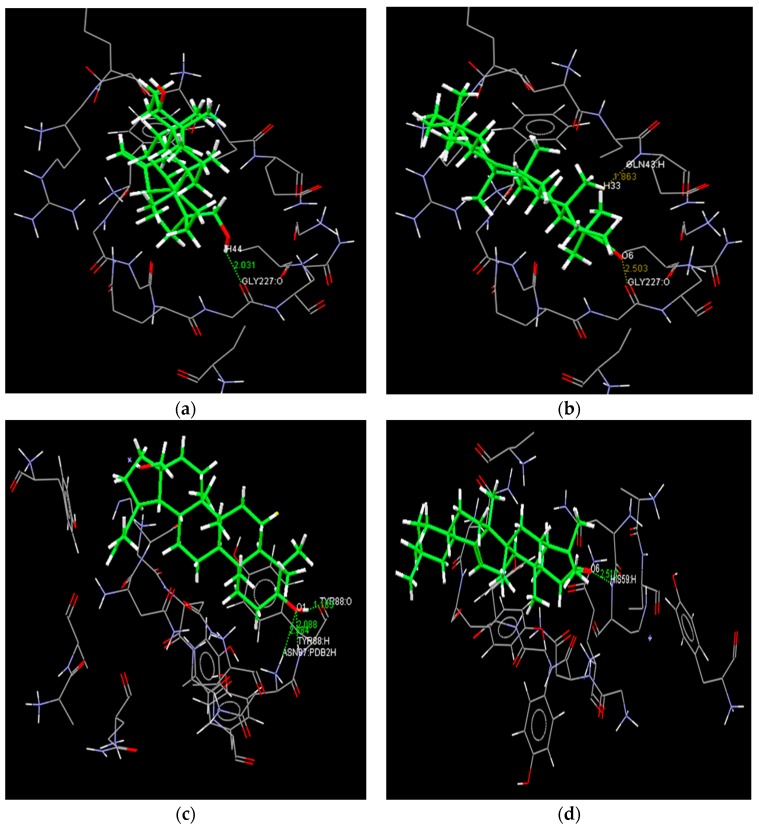
Docking poses of staphylococcal enterotoxin A (SEA) with Betulin (**a**) and 28-Norolean-12-en-3-one (**b**) compounds. A hydrogen-bond interaction is formed between H44 in Betulin and oxygen (O) in GLY227 residue of SEA (**a**); while two hydrogen-bond interactions are formed by H33 and H in 28-Norolean-12-en-3-one and O6 in GLN43 and O in GLY227 of SEA, respectively (**b**); Docking poses of staphylococcal enterotoxin B-like (SEB-L) with Betulin (**c**) and 28-Norolean-12-en-3-one (**d**) compounds. Hydrogen-bond interaction is formed by O1 in Betulin and O in TYR88 and H in ASN87 and TYR88 of SEB-L, respectively (**c**); while a hydrogen-bond interaction is formed between O6 in 28-Norolean-12-en-3-one and H in HIS59 residue of SEB-L (**d**). Residues located 5 Å from the compounds along with bond length are shown.

In order to predict the binding modes of pharmacophore models adopted from structural manipulations of the compounds, docking stimulations of the interactions were examined. Using the Molinspiration server [35], a library of 18 compounds was screened for satisfying the minimal chemical criteria for further analysis. Among these compounds, Betulin and 28-Norolean-12-en-3-one (Table 1) were selected on the basis of the criteria to satisfy the Lipinski’s Rule-of-Five [36] with zero violations for docking onto enterotoxin models. All docking measurements were carried out using Fast Rigid Exhaustive Docking (FRED) v.2.1, which is based on the Rigid Body Shape-Fitting (Open Eye Scientific Software, Santa Fe, NM, USA), and the files generated were analyzed for their binding conformations. Open Eye software has been used for docking various drug molecules with the binding site of receptors from different sources [24,37,38]. In our study, analysis was based on the free energy of binding, the lowest docked energy and the calculated RMSD values. All clusters with docking conformations to the lead molecules that showed negative binding energies were considered to have the strongest binding affinity with the enterotoxins. The docking of 28-Norolean-12-en-3-one to SEA formed a stable complex by the formation of two hydrogen bonds with GLN43 and GLY227 with a binding free energy of −83.97 kcal/mol, whereas that of Betulin to SEA showed GLY227 establishing a hydrogen bond with Betulin with a binding free energy of −147.39 kcal/mol (Table 1 and Figure 7). Likewise, the docking study of Betulin and 28-Norolean-12-en-3-one with SEB-like demonstrated that (i) two hydrogen bonds formed with ASN87 and TYR88 residues of SEB-like stabilized the complex with Betulin by a binding free energy of −139.97 kcal/mol, and (ii) HIS59 residue in the binding site of SEB-like established a hydrogen-bond interaction with 28-Norolean-12-en-3-one with a binding free energy of −88.20 kcal/mol (Table 1 and Figure 7). All the docking poses revealed that the tested ligand molecule interacts with one or two amino acids in the binding sites of SEA and SEB-like (Figure 7). Overall, on the basis of the number of hydrogen-bond interactions the present result showed that 28-Norolean-12-en-3-one acts as a good inhibitor for SEA, while Betulin is for SEB-like. Therefore, we may conclude that the interaction between the enterotoxin receptor and the ligand is a mixture of non-polar and polar types. Similarly, possible binding sites of white spot syndrome virus target protein VP26 (PDB ID: 2EDM) were searched using CASTp [39]. CASTp has also been used by many drug companies in drug discovery process to determine binding sites in a number of target molecules [40,41,42].

Betulin is a natural product, which is the main source of birch bark [43]. The potent biological activity of this compound is due to esterification, and various methods of esterification have been proposed to enhance its activity [44,45,46,47,48,49,50,51,52,53]. The anticancer activity of this compound toward human melanoma was reported decades ago [45]. Later, several studies have revealed that Betulin is not only melanoma-specific, but is also fairly active against many other cancer cell types [46,47,48]. The derivatives of Betulin have been explored for their significant properties like anti-bacterial [49], antimalarial [50], anti-inflammatory [51], anthelmintic activities [52] and anti-HIV [53]. Similarly, Noroleans are recognized as important chemical constituents of the family Lardizabalaceae, and Noroleanane triterpenoids have a wide range of bioactivities [54,55,56]. Other studies have shown that Nortriterpenoids are also cytotoxic against HCT-116, HepG2 and SGC-7901 cancer cell lines [57,58]. The biological properties of Betulin, Noroleans and their derivatives are well documented in literature; however, no reports have been available yet regarding the antitoxin activity of these compounds. Hence, the results of their antitoxin activity in the present study revealed a potential novel biological property of Betulin and Noroleans.

## 3. Materials and Methods

### 3.1. Source of Data

The amino-acid sequences of the SEA (accession number: BAF68155) and SEB-like (accession number: KR819504) were retrieved from the database of the National Center for Biotechnology Information (www.ncbi.nlm.nih.gov).

### 3.2. Generation of the 3-D Structure Using Homology Modeling

The amino-acid sequences of both enterotoxins SEA and SEB-like were blast-searched against RCSB PDB (www.rcsb.org) to identify the protein structures 1ESF and 4RGM [59]. Furthermore, each of the identified protein structures was used as a template for homology modelling using MODELLER9.17 (University of California, San Francisco, CA, USA), and the protein structure template was represented as probability density functions (PDFs) for the features restrained. The generated 3-D models were further used to actualize the homology of proteins by ideally fulfilling spatial restrictions developed from the alignment [60].

### 3.3. Molecular Dynamics

The generated 3-D structures were further improved with a blend of sub-atomic elements and equilibration strategies by NAMD programming for lipids and proteins, along with Transferable Intermolecular Potential with 3 Points model for water [61]. The security extending, edge bowing, torsional and other compelling field parameters for SEA and SEB-like were specifically connected to guarantee redress hybridization using Chemistry at Harvard Macromolecular Mechanics 27 (CHARMM27) driven from the field protein parameters [62,63]. The vitality of the structures was upgraded for side chains, and dissolvable with 100,000-stage minimization to remove any bad contacts with 12 Å cutoff (switching function starts at 10 Å) for van der Waals interactions. A mix time-venture of 2 femtoseconds (fs) interim was used, allowing a different time-venturing calculation in which interaction of all covalent bonds involving hydrogens were figured at each time step [64]. Short-run non-fortified communications were registered for consistent time step and long-extend electrostatic powers were processed for every four-time step. The combined rundown of the non-fortified communications was recalculated for every ten-time venture with a couple list separation of 13.5 Å. The short scope of non-reinforced communications was within 12 Å, and smoothing procedure was used for van der Waals associations at the 10 Å cutoff. An equilibrated framework was recreated for two ps with a 500 kcal/mol/Å limitation on the protein spine under constant pressure of one atmospheric pressure (atm) steady weight and constant temperature of 310 Kelvin (K) (typical weight and temperature). The Langevin damping coefficient was set to 20 ps unless generally stated [65].

The structure with the most minimal vitality and steady low RMSD was utilized for further reviews. The final refined 3-D models of SEA and SEB-like were dissected by using Ramachandran plot calculations computed with the RAMPAGE (http://mordred.bioc.cam.ac.uk/~rapper/rampage.php) program [26]. Subsequently, the ERRAT diagram (Structure Evaluation server, http://services.mbi.ucla.edu/ERRAT/) and PROCHECK program (http://services.mbi.ucla.edu/PROCHECK) were utilized to perform environmental profiling for deciding stereo substance nature of the protein structures [66].

### 3.4. Binding Site Identification

Binding sites of SEA and SEB-like were identified using the CASTp server, (http://sts.bioe.uic.edu/castp/). CASTp identifies all feasible pockets in the targeted protein structure and measures both the solvent-accessible surface area (Lee-Richards molecular surface) and solvent-excluded molecular surface (MS, Connolly surface) area, and pockets and pocket mouth openings, as well as cavities.

### 3.5. Substrate Docking

The chemical structures of Betulin and 28-Norolean-12-en-3-one compounds were built with ChemSketch software suite v.15.01 (ACD/Structure Elucidator, Advanced Chemistry Development, Inc., Toronto, ON, Canada, http://www.acdlabs.com) and optimized using Molinspiration server (http://www.molinspiration.com) [35,39]. Ligands were docked with the binding sites of SEA and SEB-like by extremely FRED v.2.1 (Open Eye Scientific Software, Santa Fe, NM, USA). FRED enabled us to implement multi conformer docking in two steps. First, a conformational screening of the ligand was conducted, and then all relevant low-energy conformations were rigidly placed in the binding sites. These two steps allowed the formation of the rigid structure by the translational degree of freedom. The FRED process was used for the series of shape-based filters, and the default scoring function was assigned based on the Gaussian shape fitting [67].

## 4. Conclusions

In our study, the stable 3-D SEA and SEB-like models were constructed and further used for docking with the inhibitors Betulin and 28-Norolean-12-en-3-one. Docking results revealed several important amino-acid residues in the binding sites of SEA and SEB-like which play essential role in maintenance of their conformation and are directly implicated in substrate binding. The interactions between the inhibitors and the binding sites of SEA and SEB-like projected in this study are useful for the in-depth understanding of the binding mechanisms of inhibitors and active domain of enterotoxins. The bioinformatic tools described in this study are simple but highly effective and robust. The results revealed that the predicted conformations are in close agreement with the experimental structures available in literature. The method used in the current study provides a platform for in silico validation and control of *S. aureus* infection, resulting in effective control of food poisoning-related effects.

## Figures and Tables

**Table 1:** Ligand interactions with enterotoxins along with energy (kcal/mol) values.

Compound Name	PubChem CID	Molecular Formula	Molecular Weight (g/mol)	Compound Structure	Staphylococcal Enterotoxin (SE) in Interaction	Free Energy (kcal/mol)
Betulin	72326	C_30_H_50_O_2_	442.728	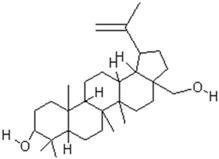	SEA	−147.39
					SEB-like	−139.97
28-Norolean-12-en-3-one	101616676	C_29_H_46_O	410.686	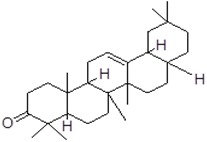	SEA	−83.97
					SEB-like	−88.20

## Supplementary Materials

Supplementary materials can be found at www.mdpi.com/1422-0067/19/1/133/s1.

## Abbreviations

3-D	Three-Dimensional
atm	Atmospheric Pressure
CASTp	Computer Atlas of Surface Topology of Proteins
CHARMM	Chemistry at Harvard Macromolecular Mechanics
FRED	Fast Rigid Exhaustive Docking
K	Kelvin
MS	Molecular Surface
NAMD	NAnoscale Molecular Dynamics
NCBI	National Center for Biotechnology Information
PDB	Protein Data Bank
PDFs	Probability Functions
ps and fs	Pico- and Femtoseconds
RCSB	Research Collaboratory for Structural Bioinformatics
RMSD	Root-Mean-Square Deviation
SEA	Staphylococcal Enterotoxin A
SEB	Staphylococcal Enterotoxin B
SEs	Staphylococcal Enterotoxins
