# Supplementary material for: Computational Modeling of the Staphylococcal Enterotoxins and Their Interaction with Natural Antitoxin Compounds
Source: Int J Mol Sci. 2018 Jan 3;19(1):133. doi: 10.3390/ijms19010133 (PMC5796082; doi:10.3390/ijms19010133)
Supplement: Supplementary file 1 [file ijms-19-00133-s001.pdf]

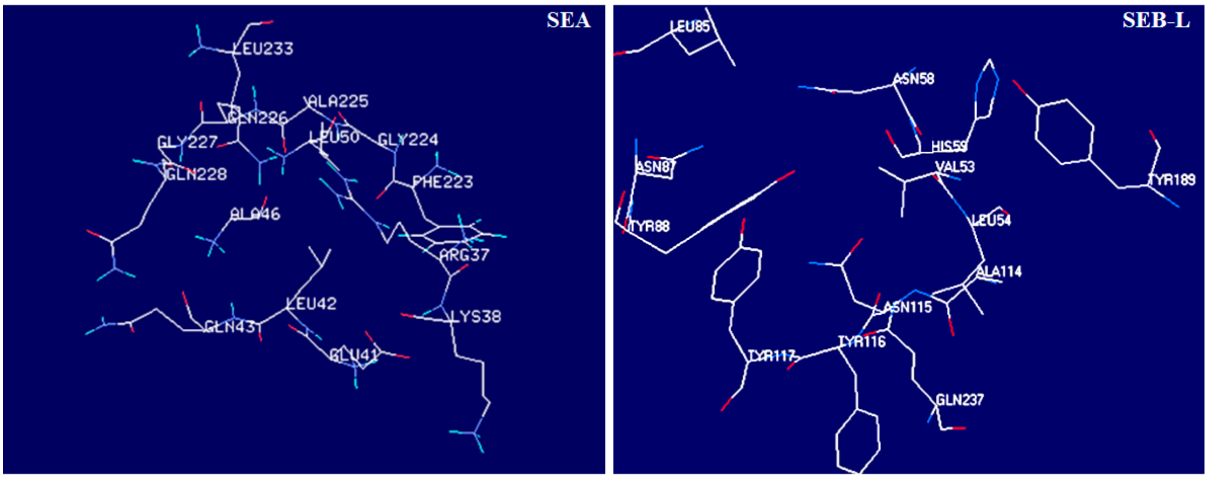

Supplementary Figure S1. Amino acids present at the binding sites of the staphylococcal enterotoxin A (SEA) and staphylococcal enterotoxin B-like (SEB-L) as detected by the Computer Atlas of Surface Topology of Proteins (CASTp) are shown.
